# Clinical characteristics and therapeutic strategy for patients with spontaneous isolated abdominal aortic dissection

**DOI:** 10.3389/fcvm.2023.1214377

**Published:** 2023-08-25

**Authors:** Zhengde Zhao, Jiawei Liu, Yunchong Liu, Kan Huang, Mian Wang, Ridong Wu, Zuojun Hu, Chen Yao, Zilun Li, Guangqi Chang

**Affiliations:** ^1^ Division of Vascular Surgery, The First Affiliated Hospital of Sun Yat-sen University, Guangzhou, China; ^2^ Private Medical Service & Healthcare Center, The First Affiliated Hospital of Sun Yat-sen University, Guangzhou, China

**Keywords:** spontaneous isolated abdominal aortic dissection, manifestation, conservative therapy, endovascular aortic repair, outcome

## Abstract

**Objective:**

Spontaneous isolated abdominal aortic dissection (SIAAD) is a rare aortic emergency and not yet fully understood. This study aims to report the characteristics and treatments of 31 patients with SIAAD in the past 12 years.

**Methods:**

A total of 31 consecutive patients with SIAAD between 2010 and 2022 were included. The clinical manifestations, treatment strategies, and outcomes were reviewed. Following the SVS/STS reporting standard, we compared the clinical characteristics with different locations of primary entry, or different numbers of dissected zones. Furthermore, we compared the effects of surgical and conservative therapies on the outcome during the follow-up.

**Results:**

Among the 31 patients with SIAAD, 16 (51.6%) were in the acute phase on admission. The primary entry of SIAAD was mainly located in Zone 9 (67.7%). Most patient presented with dissection involving 1 or 2 aortic zones (61.3%). In addition, 35.5% and 64.5% of SIAADs involved the visceral and iliac arteries, respectively. Compared with asymptomatic SIAADs, the symptomatic ones had longer dissection lengths (*P* = 0.008) and tended to involve iliac artery more frequently (*P* = 0.098). There were differences in the number of dissected aortic zones (*P* = 0.005) among patients with primary entry located in Zone 5 (Supraceliac aorta), Zone 6–8 (Paravisceral aorta) and Zone 9 (Infrarenal aorta). The involvement of visceral artery (*P* = 0.039) and iliac artery (*P* = 0.006) was significantly different between the subgroups of SIAAD involving one, two, and three or more aortic zones. The cumulative incidence of adverse false lumen progression events was significantly lower (*P *= 0.000) and the rate of false lumen thrombogenesis or disappearance was higher in patients receiving surgery (*P* = 0.001). The cumulative all-cause mortality was 9.7% at 1-year, and 19.7% at 5-year, with no significant difference between surgical and conservative therapies.

**Conclusions:**

Clinical features of SIAAD vary depending on the location of the primary entry and the number of dissected aortic zones. Although surgery was not associated with a lower all-cause mortality compared with conservative therapy, it was associated with a lower incidence of adverse false lumen progression and a higher rate of aortic remodeling.

## Introduction

Aortic dissection (AD) is a rare but fatal cardiovascular emergency mainly involving the ascending and descending aorta. Isolated abdominal aortic dissection accounts for 1%–4% of AD (1–3), with lesions limited to the abdominal aorta, as initially reported by Shekelton in 1822 (4). Currently, only small numbers of spontaneous isolated abdominal aortic dissection (SIAAD) were reported in several centers (5–10), and the characteristics and prognosis of SIAAD remain incompletely understood.

The symptoms of SIAAD are atypical, with abdominal or back pain as the main manifestation, while few patients present with limb ischemia. Notably, 10%–15% of the SIAAD may lead to aortic rupture (11, 12). Treatments of SIAAD include open surgery, endovascular aortic repair (EVAR), and conservative therapy. A meta-analysis of 491 cases showed that surgical treatments including open surgery and EVAR does not lower the early and late mortality compared to the conservative management (13). However, a more recent report argued that operation facilitated aortic remodeling and reduced adverse false lumen progression (14). The optimal treatment of SIAAD remains controversial.

This study aims to report the characteristics of 31 patients with SIAAD and to summarize the treatment outcomes in the patients managed with conservative or surgical treatments.

## Methods

### Enrollment of patients

SIAAD was defined as a spontaneous AD confined to the abdominal aorta below the aortic fissure and above the aortic bifurcation, regardless of its extension to visceral or iliac arteries. Patients with SIAAD were retrieved in the electronic medical record system, with exclusion of AD secondary to iatrogenic or traumatic causes, and intermural aortic hematomas or ulcers. The study was approved by the Ethics Committee for Clinical Research and Laboratory Animal Trials of the First Affiliated Hospital of Sun Yat-sen University, and all patients were informed and exempted from the informed consent due to the retrospective design.

### Data collection and definition

Demographic data, stages, symptoms, comorbidity and risk factors were collected through the electronic medical record system. The stages of acute, subacute and chronic phase were defined as 1–14 days, 15–90 days and >90 days from the onset of symptoms, respectively. Aortic features, including direction of the entry tear (12–3 o’clock; 3–6 o’clock; 6–9 o’clock; 9–12 o’clock), length of the dissection, total aortic diameter, true lumen diameter, false lumen diameter, status of false lumen (patent, partial thrombosis, complete thrombosis), involvement of visceral or iliac artery, coexisting abdominal aortic aneurysm (AAA), were evaluated based on the imaging.

The abdominal aorta and iliac arteries can be divided into zones 5–11 (Supplementary Table S1), according to the Society for Vascular Surgery (SVS)/Society for Thoracic Surgery (STS) (SVS/STS) reporting standard published in 2020 (15). In this study, the SIAAD patients with lesion confined to the abdominal aorta were classified into three subgroups based on the location of primary entry tear according to SVS/STS reporting standard: Group 1 with primary entry tear located in Zone 5 (from superior border of T12 to the celiac trunk artery), Group 2 with primary entry tear located between Zone 6 and Zone 8 (from the celiac trunk artery to the lowest renal artery), and Group 3 with primary entry tear located between Zone 9 (from the lowest renal artery to the aorto-iliac bifurcation). In addition, patients were categorized into three subgroups based on the initial number of dissected zones: Group A with dissection involving one aortic zone, Group B with dissection involving two aortic zones, and Group C with dissection involving three or more aortic zones.

### Treatment strategy

Treatment strategies were determined based on patients' clinical and anatomical features. Criteria for surgical treatment include: (1) presence of aortic rupture or pending rupture, (2) presence of organ or limb ischemia, (3) abdominal aortic diameter ≥30 mm, (4) symptoms did not relieve after standard medical treatment. For patients appropriate for endovascular repair, aortography was performed to determine the primary entry tear site, and the location and extent of the dissection. Endovascular repair including standard EVAR, EVAR with chimney technique to reconstruct visceral arteries, straight stent graft to cover the tears, kissing stent graft technique (16), and hybrid strategy was applied to individual patients depending on the anatomical features. Patients without surgical indications were treated with conservative therapy including strict control of blood pressure and heart rate.

### Follow-up

The patients were followed up by CTA at 1 month, 3 months, 6 months and 12 months after surgery or discharge with conservative therapy, and annually thereafter. Symptoms, complications, and survivals were recorded, while progression of the false lumen was evaluated by the CTA.

### Statistical analysis

Normal distribution variables were expressed as mean ± standard deviation, and comparisons were performed using *t*-test or ANOVA. Non-normal distribution variables were expressed as median (IQR, P25–P75) and compared using the Mann–Whitney *U* test or Kruskal–Wallis H test. Kaplan–Meier analysis was used to compare the cumulative outcomes during follow-up, and log-rank tests were used for comparisons within groups. All statistical analyses were performed using SPSS 26.0 software, with *P *< 0.05 being considered as statistically significant.

## Results

### Demographics

From 2010 to 2022, a total of 31 patients with SIAAD were included in the study. The mean age was 57.8 ± 2.3 years. On admission, 16 (51.6%), 4 (12.9%), and 11 (35.5%) patients were in the acute, subacute, and chronic phases, respectively. Hypertension were found in 71.0% of SIAAD patients. Only 19.4% and 12.9% patients presented with diabetes and coronary artery disease, respectively. Five cases had a history of smoking. One patient had a connective tissue disease (Table 1).

**Table 1:** Patient demographic and comorbidities data.

Feature	
Age, years	57.8 ± 2.3
Sex	
Female	11 (35.5%)
Male	20 (64.5%)
Stage	
Acute	16 (51.6%)
Subacute	4 (12.9%)
Chronic	11 (35.5%)
Hypertension	22 (71.0%)
Diabetes	6 (19.4%)
Coronary heart disease	4 (12.9%)
Renal insufficiency	6 (19.4%)
Renal cyst	0
Chronic obstructive pulmonary disease	4 (12.9%)
History of smoking	
Current Smoker	4 (12.9%)
Past smoker	1 (3.2%)
Not smoker	26 (83.9%)
Vasculitis	0
Connective tissue disease	1 (3.2%)

### Clinical presentation

Of the 31 patients with SIAAD, 25 (80.6%) were symptomatic, with abdominal pain (17 cases, 68.0%) and back pain (8 cases, 32.0%) as the main symptoms. The primary entry tear was mainly located in Zone 9 (21 cases, 67.7%), with proximal extent mainly in Zone 9 (22 cases, 71.0%) and distal extent mostly in Zone 10–11 (21 cases, 67.8%). There were 3 (9.7%), 8 (25.8%), and 20 (64.5%) cases with dissection involving the superior mesenteric artery, renal artery, and iliac artery, respectively (Table 2).

**Table 2:** Symptom and morphologic features at the initial presentation in patients with SIAAD.

Feature	
Symptom	
Symptomatic	25 (80.6%)
Abdominal pain	17 (68.0%)
Back pain	8 (32.0%)
Chest pain	1 (4.0%)
Foot coldness	2 (8.0%)
Asymptomatic	6 (19.4%)
Length of Dissection, mm	47.7 (31.8, 109.1)
AD diameter, mm	26.0 (21.9, 35.4)
False Lumen diameter, mm	13.8 (10.8, 17.2)
True Lumen diameter, mm	12.0 (10.7, 15.2)
Primary entry tear	
Zone 5	4 (12.9%)
Zone 7	1 (3.2%)
Zone 8	5 (16.1%)
Zone 9	21 (67.7%)
Proximal extent	
Zone 5	6 (19.3%)
Zone 8	3 (9.7%)
Zone 9	22 (71.0%)
Distal extent	
Zone 6	1 (3.2%)
Zone 9	9 (29.0%)
Zone 10	11 (35.5%)
Zone 11	10 (32.3%)
Initial no. zones dissected	
1	7 (22.5%)
2	12 (38.7%)
≥3	12 (38.7%)
Direction of the entry site of AD	
12–3 o’clock	8 (25.8%)
3–6 o’clock	10 (32.3%)
6–9 o’clock	4 (12.9%)
9–12 o’clock	7 (22.6%)
Initial false lumen patency	
Patent	20 (64.5%)
Partial thrombosis	11 (35.5%)
Complete thrombosis	0
Visceral artery involvement	
Celiac trunk artery	0
Superior mesenteric artery	3 (9.7%)
Renal arteries	8 (25.8%)
Iliac artery involvement	20 (64.5%)
Coexisting AAA	6 (19.4%)

AD, aortic dissection; AAA, abdominal aortic aneurysm.

Compared with asymptomatic SIAAD, patients with symptomatic SIAAD had younger ages (56.7 ± 2.7 vs. 62.3 ± 4.7) and higher percentage of female gender (40.0% vs. 16.7%). Visceral artery involvement (32.0% vs. 0%) and iliac artery involvement (72.0% vs. 33.3%) were higher in symptomatic patients than that in asymptomatic patients, although the results did not reach a statistical significance. Length of dissection was significantly longer in symptomatic patients compared to asymptomatic patients (*P* = 0.008, Table 3), and the number of dissected zones in symptomatic patients tended to be larger than that in asymptomatic patients (Table 3). All asymptomatic patients had primary entry tears in Zone 9, while symptomatic patients had primary entry tears in multiple aortic zones.

**Table 3:** Comparison of patients’ characteristics and morphologic features by the symptom status.

Symptom status	Asymptomatic	Symptomatic	*P*-value
No. of patients	6	25	
Age, years (Mean ± SD)	62.3 ± 4.7	56.7 ± 2.7	0.326
Sex, female	1 (16.7%)	10 (40.0%)	0.383
Hypertension	5 (83.3%)	17 (68.0%)	0.642
Aortic calcification	5 (83.3%)	14 (56.0%)	0.488
Visceral artery involvement	0	8 (32.0%)	0.137
Iliac artery involvement	2 (33.3%)	18 (72.0%)	0.098
Initial Status of False Lumen, Partial thrombosis	1 (16.7%)	10 (40.0%)	0.383
Initial no. zones dissected [Median (IQR)]	1.5 (1, 2)	2 (2, 3)	0.174
Primary entry tear			0.696
Zone 5	0	4	
Zone 7	0	1	
Zone 8	0	4	
Zone 9	6	16	
Coexisting AAA	2 (33.3%)	4 (16%)	0.567
D-D, mg/L [Median (IQR)]	0.7 (0.6, 1.5)	1.5 (0.8, 3.5)	0.355
Length of AD, mm [Median (IQR)]	31.4 (30.3,40.0)	86.6 (41.0, 113.6)	0.008
AD diameter, mm [Median (IQR)]	26.5 (21.9, 35.5)	26.6 (21.6, 33.8)	0.979
Coexisting AAA	37.0 (35.5, 38.4)	56.9 (44.8, 70.3)	0.530
Not Coexisting AAA	24.0 (21.2, 26.5)	24.4 (21.6, 28.1)	0.667
True Lumen diameter, mm [Median (IQR)]	11.5 (10.9, 13.6)	13.4(10.1, 15.7)	0.854
Coexisting AAA	16.0 (12.0, 19.9)	17.6 (14.3, 32.1)	0.530
Not Coexisting AAA	10.9 (10.8, 12.3)	12.9 (8.6, 16.7)	0.725
False Lumen diameter, mm [Median (IQR)]	12.7 (10.8,16.0)	14.6 (12.4, 18.3)	0.813
Coexisting AAA	19.3 (13.9, 24.7)	28.9 (16.8, 38.8)	0.800
Not Coexisting AAA	11.2 (9.8, 14.88)	12.9 (8.6, 16.7)	0.611

AD, aortic dissection; SD, standard deviation; IQR, interquartile range; AAA, abdominal aortic aneurysm; D-D, D dimer.

None of the patients with primary entry tears in Group 3 (Zone 9) had a retrograde extension to the aorta in Zone 5–8. Visceral artery involvement was present in the patients with primary entry tears in either Group 1 (Zone 5) or Group 2 (Zone 6–8). The length of dissection tended to be longer in the SIAAD with primary entry tears in Group 2 (Zone 6–8) than Group1 (Zone 5) and Group 3 (Zone 9) (*P* = 0.051) (Table 4). Moreover, the initial number of dissected zones showed a significant difference among the three subgroups (*P* = 0.005). Comparison between subgroups showed that the initial number of dissected zones for patients with primary entry tears in Group 1 (Zone 5) and Group 2 (Zone 6–8) was both significantly greater than Group 3 (Zone 9) (*P *= 0.030, *P *= 0.039) (Table 4).

**Table 4:** Comparison of patients’ characteristics and morphologic features by the location of primary entry tear.

Location of primary entry tear	Group 1 (Zone 5)	Group 2 (Zone 6–8)	Group 3 (Zone 9)	*P*-value
No. of patients	4	6	21	
Age, years (Mean ± SD)	62.0 ± 6.4	47.3 ± 3.5	59.9 ± 2.9	0.104
Sex, female	2 (50.0%)	1 (16.7%)	8 (38.1%)	0.617
Symptomatic	3 (75.0%)	6 (100%)	16 (76.2%)	0.510
Hypertension	4 (100%)	4 (66.7%)	14 (66.7%)	0.499
Aortic calcification	3 (75%)	2 (33.3%)	14 (66.7%)	0.506
Visceral artery involvement	2 (50.0%)^a^	6 (100%)^b^	0^a,b^	0.000
Iliac artery involvement	1 (25.0%)	5 (83.3%)	14 (66.7%)	0.193
Initial Status of False Lumen, Partial thrombosis	2 (50%)	4 (66%)	5 (23.8%)	0.136
Initial no. zones dissected [median (IQR)]	5.5 (3.0,7.0)^c^	5.0 (2.5,7.0)^d^	2.0 (1.0,2.0)^c,d^	0.005
D-D, mg/L [Median, (IQR)]	2.9 (0.9,5.8)	3.7 (2.1,6.8)	0.9 (0.6,1.7)	0.112
Length of AD, mm [Median (IQR)]	94.7 (29.2,170.8)	119.6 (81.6,189.1)	47.2 (31.0,91.6)	0.051
Coexisting AAA	0	1	5	0.522
AD diameter, mm [Median (IQR)]	27.4 (25.5, 33.3)	28.6 (24.8, 39.1)	26.0 (21.5, 35.4)	0.644
Coexisting AAA	–	–	54.1 (35.5, 59.7)	0.343
Not Coexisting AAA	26.6 (25.5, 27.4)	28.0 (24.3, 29.2)	23.0 (21.1, 26.0)	0.153
True Lumen diameter, mm [Median (IQR)]	13.2 (11.7, 17.1)	12.7 (8.7, 15.8)	11.2 (10.6, 15.2)	0.431
Coexisting AAA	–	–	19.9 (15.1, 20.0)	0.538
Not Coexisting AAA	14.3 (12.8, 17.1)	10.7 (7.4, 14.6)	10.9 (10.1,13.4)	0.247
False Lumen diameter, mm [Median (IQR)]	13.4 (10.2, 19.3)	14.7 (10.3, 24.3)	14.6 (11.5,17.2)	0.973
Coexisting AAA	–	–	19.8 (13.9, 38.0)	0.172
Not Coexisting AAA	12.9 (10.2, 13.4)	12.8 (12.7, 16.7)	12.0 (9.0, 16.4)	0.746

AD, aortic dissection; SD, standard deviation; IQR, interquartile range; AAA, abdominal aortic aneurysm; D-D, D dimer.

^a^ *P-*value <0.05, comparison between Group 1 (Zone 5) and Group 3 (Zone 9).

^b^ *P*-value <0.05, comparison between Group 2 (Zone 6–8) and Group 3 (Zone 9).

^c^ *P-*value = 0.030, comparison between Group 1 (Zone 5) and Group 3 (Zone 9).

^d^ *P*-value = 0.039, comparison between Group 1 (Zone 6–8) and Group 3 (Zone 9).

The distribution of visceral arterial involvement was significantly different among the three subgroups (Group A, Zone = 1; Group B, Zone = 2; Group C, Zone ≥3) (*P* = 0.039), but the dissection extent of SIAAD in the Group A (Zone = 1) was all confined to Zone 9 (Table 5). For iliac artery involvement, it occurred in 83.3% cases in the Group C (Zone ≥3), 75.0% cases in the Group B (Zone =2), and only 14.3% cases in the Group A (Zone =1), with a significantly different distribution among the three subgroups (*P *= 0.006), and the incidence of iliac artery involvement in the Group B (Zone = 2) and Group C (Zone ≥3) was both significantly higher than that in Group A (Zone = 1) (Table 5). In addition, the false lumen diameter tended to be larger in the Group C (Zone ≥3) than both the Group A (Zone = 1) and Group B (Zone = 2) (*P *= 0.102). After excluding the cases coexisting with AAA, there was a significant difference in false lumen diameter among the three subgroups (*P *= 0.025), and the false lumen diameter was significantly larger in the Group C (Zone ≥3) than that in the Group B (Zone = 2) (Table 5).

**Table 5:** Comparison of patients’ characteristics and morphologic features by initial number of aortic zones dissected.

Initial no. zones dissected	Group A (Zone = 1)	Group B (Zone = 2)	Group C (Zone ≥3)	*P-*value
No. of patients	7	12	12	
Age, years (Mean ± SD)	62.7 ± 4.6	58.3 ± 4.1	54.5 ± 3.6	0.423
Sex, female	1 (14.3%)	5 (41.7%)	5 (41.7%)	0.556
Symptomatic	4 (57.1%)	10 (83.3%)	11 (91.7%)	0.183
Hypertension	6 (85.7%)	7 (58.3%)	9 (75%)	0.605
Aortic calcification	5 (71.4%)	8 (66.7%)	6 (50.0%)	0.606
Visceral artery involvement	0	2 (16.7%)	6 (50.0%)	0.039
Iliac artery involvement	1 (14.3%)^a^	9 (75.0%)^b^	10 (83.3%)^a,b^	0.006
Initial status of False Lumen, Partial thrombosis	2 (28.6%)	3 (25%)	6 (50.0%)	0.504
Coexisting AAA	1 (14.3%)	2 (16.7%)	3 (25%)	1.000
D-D, mg/L, [Median, (IQR)]	0.6 (0.4, 0.9)	1.4 (0.7,2.9)	2.0 (1.5,4.3)	0.071
Length of AD [Median, (IQR)]	35.5 (30.0, 50.1)^c^	37.0 (30.3,51.4)^d^	113.6 (86.6, 159.3)^c,d^	0.008
AD diameter, mm [Median (IQR)]	24.0 (21.5, 26.0)	26.6 (23.0, 28.0)	30.7 (24.4, 38.2)	0.230
Coexisting AAA	–	67.5 (54.1, 80.8)	36.9 (38.4, 49.1)	0.343
Not Coexisting AAA	23.1(21.5, 26.0)	24.2 (20.7, 26.8)	28.1 (24.3, 32.2)	0.187
True Lumen diameter, mm [Median (IQR)]	10.9 (9.5, 13.6)	15.1 (10.9, 16.9)	12.7 (10.7, 14.6)	0.423
Coexisting AAA	–	29.6 (15.1, 44.1)	13.4 (12.7, 16.7)	0.538
Not Coexisting AAA	10.9 (9.5, 11.1)	12.2 (10.8, 16.6)	11.3 (7.9, 14.6)	0.390
False Lumen diameter, mm [Median (IQR)]	12.7 (10.8, 14.1)	12.0 (7.8, 17.2)	17.0 (13.8, 19.8)	0.102
Coexisting AAA	–	38.8 (38.0, 39.6)	19.8 (16.8, 22.3)	0.172
Not Coexisting AAA	11.2(8.6, 14.1)	8.6 (7.1, 14.0)^e^	14.6 (12.9, 17.2)^e^	0.025

AD, aortic dissection; SD, standard deviation; IQR, interquartile range; AAA, abdominal aortic aneurysm; D-D, D dimer.

^a^ *P*-value <0.05, comparison between Group A (Zone = 1) and Group C (Zone≥3).

^b^ *P*-value <0.05, comparison between Group B (Zone = 2) and Group C (Zone ≥3).

^c^ *P*-value = 0.032, comparison between Group A (Zone = 1) and Group C (Zone ≥3).

^d^ *P*-value = 0.024, comparison between Group B (Zone = 2) and Group C (Zone ≥3).

^e^ *P*-value = 0.027, comparison between Group B (Zone = 2) and Group C (Zone ≥3).

### Treatment outcomes

Eight patients received conservative treatment, while 23 patients underwent surgical treatment. including one hybrid surgery and 22 endovascular repair. Type I–III endoleaks were observed in 5, 1, and 1 patients in completion angiography, respectively (Supplementary Table S2). For one case involving Zone 5-Zone 8, the celiac trunk artery was intentionally covered to gain a sufficient landing zone. For patients with lesions involving all the paravisceral and infrarenal aorta (Zone 5-Zone 11), one was treated by straight stent graft combined with chimney stenting, one with entry tear in a collateral renal artery was sealed with aortic stent graft, and one with small entry tear in renal artery was left untreated and the infrarenal lesion was treated with aortic stent graft. For lesions involving Zone 9, straight aortic stent grafts were used in 2 cases, and bifurcated stent grafts were used for lesions near aortic bifurcation or involving the iliac artery in 3 cases. For SIAAD involving Zone 9–11, bifurcated stent graft was applied in 7 cases. When the diameter of the true lumen at the aortic bifurcation was less than 15 mm, straight stent graft combined with unilateral iliac stent graft was used in 1 case with unilateral iliac artery involved, while the kissing stent technique was used in 3 cases with bilateral iliac artery involved.

There was no significant difference in the length of hospital stay between conservative and surgical treatments. Furthermore, no major cardiovascular events or aortic rupture occurred during hospitalization after either treatment (Table 6). One patient receiving surgery developed acute renal injury, which fully recovered after rehydration therapy (Table 6). No patient died within 30 days for either treatment.

**Table 6:** Outcome of different treatment strategies.

	Surgical (*n* = 23)	Conservative (*n* = 8)	*P*-value
Within hospitalization			
Length of hospital stay, day (Mean ± SD)	16.5 ± 2.3	12.1 ± 2.0	0.317
Major CVD events	0	0	–
Aortic Rupture	0	0	–
Acute kidney injury	1	0	–
Rehospitalization or death within 30 days	0	0	–
Follow-up			
False lumen enlargement	0	2 (25%)	0.060
Longitudinal progression	1 (4.3%)	2 (25%)	0.156
False lumen thrombogenesis or disappearance	19 (82.6%)	1 (12.5%)	0.001
Later aortic intervention	1 (4.3%)^a^	3 (37.5%)^b^	0.043
All-cause mortality	2 (8.7%)	1 (12.5%)	1.000

SD, standard deviation; CVD, coronary artery atherosclerotic heart disease.

^a^ Reason for intervention: Persistent type II endoleaks accompanied by false lumen enlargement and abdominal dull pain for 1 year.

^b^ Reason for intervention: Patient 1: False lumen enlargement; Patient 2: Distal progression; Patient 3: Proximal progression.

**Figure 1:**
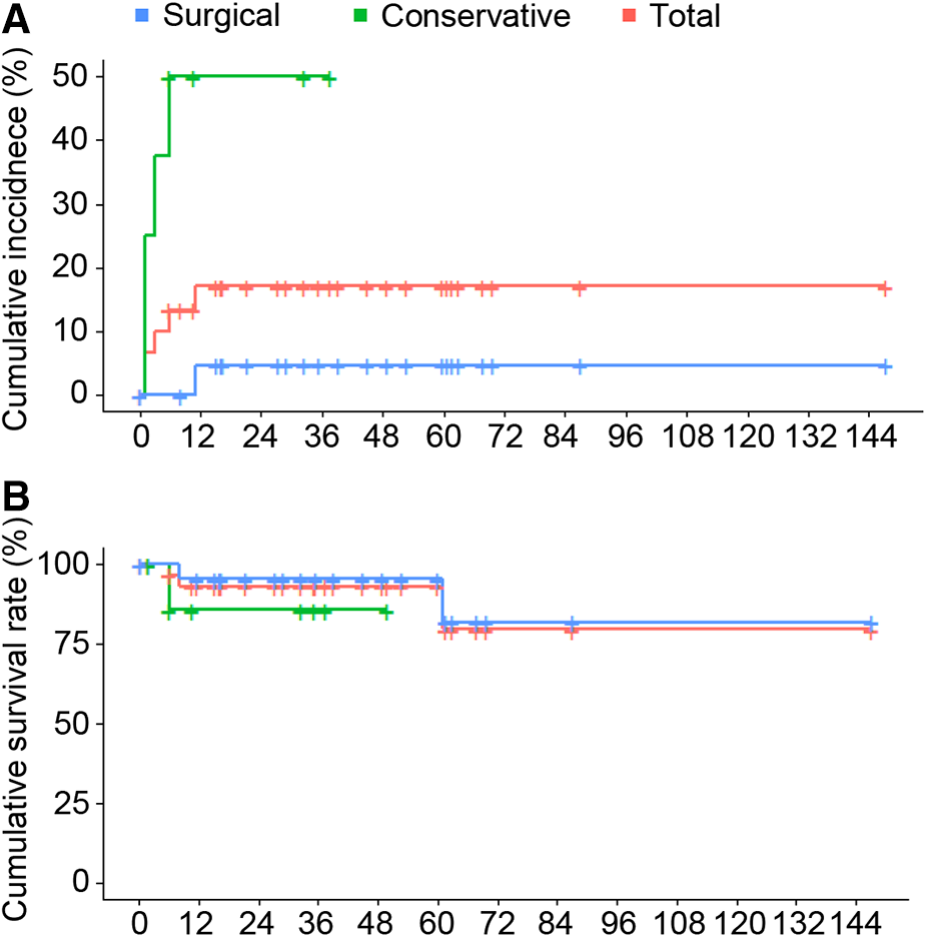
(**A**) Kaplan–Meier curves of the cumulative incidence of adverse aortic pathology during the follow-up of conservative versus surgical treatment in patients with SIAAD. (Log-Rank test: *P-*value* *= 0.000). (**B**) Kaplan–Meier curve of cumulative survival rate of conservative versus surgical treatment in SIAAD patients. (Log-Rank test: *P*-value* *= 0.320).

All patients were followed up with a median time of 37.5 months (IQR, 18.9–61.4 months). During the follow-up period, two cases (25%) developed false lumen enlargement and two cases (25.0%) developed longitudinal progression in patients receiving conservative treatment. In contrast, no false lumen enlargement and one case (4.3%) with longitudinal progression were observed in patients receiving surgery (Table 6). The Kaplan-Meier analysis in Figure 1A showed the cumulative incidence of adverse false lumen progression in patients receiving surgery was significantly lower than that in patients receiving conservative treatment (*P* = 0.000). The rate of false lumen thrombogenesis or disappearance was higher (82.6% vs. 12.5%, *P* = 0.001), while the aortic intervention rate was lower (4.3% vs. 37.5%, *P* = 0.043) in patients receiving surgery compared to patients receiving conservative treatment. In Figure 1B, the cumulative all-cause mortality was 9.5% at 1 year, and 19.5% at 5 years. No significant difference was found between surgical and conservative therapies (*P* = 0.320). In patients treated by surgery, one died of chronic heart failure 8 months after surgery, and one died of myocardial infarction 5 years later. In patients receiving conservative treatment, one died 6 months after discharge from hospital for unknown reason.

## Discussion

This study described the clinical characteristics and treatment outcomes of 32 patients with SIAAD in a single center. Symptomatic patients had longer dissection length and were more likely to involve the iliac artery. SIAAD with entry tear in Zone 6-Zone 8 tended to have a longer dissection length and involve much more aortic zones, and proned to involve visceral arteries or iliac artery. Different treatment options did not affect the incidence of adverse events and all-cause mortality within hospitalization. Surgical treatment was associated with a lower incidence of false lumen progression and a higher rate of false lumen thrombosis and aortic remodeling compared with conservative treatment.

Symptoms are important for identifying SIAAD and may vary from asymptomatic to abdominal pain, back pain, chest pain, or foot coldness, depending on the extent of SIAAD. According to previous studies including the International Registry of Acute Aortic Dissection (IRAD) study, 21%–72% patients with SIAAD were symptomatic (5, 13, 17, 18). Of note, 81.3% patients were symptomatic in this study. Identification of asymptomatic patients mainly relies on the use of enhanced CT in patients with various abdominal symptoms. In this study, a total of 6 asymptomatic cases were found under examination for abdominal diseases. SIAAD should be suspected in the presence of symptoms such as abdominal pain, limb or visceral ischemia. In our study, symptomatic SIAAD had a longer length of dissection and was more likely to involve the iliac artery.

Morphological features of SIAAD can be updated and renewed in accordance with SVS/STS guidelines. Traditionally, SIAAD was divided into the infrarenal and suprarenal types to guide the choice of open surgery. New classifications composed of supraceliac, paravisceral and infrarenal SIAAD have been proposed, but the definition varied (7, 9). SVS/STS reporting standard provided a detailed description of anatomical aortic zones (15), which could help us renew the recognition of SIAAD morphological features. Consistent with previous reports, female gender, presence of symptoms, and visceral artery involvement were more likely to occur in patients with suprarenal (Zone 5–8) SIAAD (9). In addition, we found that the SIAAD with entry tear in Zone 9 were less extensive than those with entry tear in Zone 5–8 in terms of dissection length and number of dissected aortic zones, similar to previous studies (7). Meanwhile, none of patients with primary entry tear in Zone 9 had retrograde tears proximal to suprarenal aorta, suggesting that SIAAD with more distal primary entry tears may have less severe lesions. In addition, the false lumen diameter was larger in patients with the number of dissected aortic Zones ≥3 compared to 1 or 2. The results remained consistent after excluding cases with AAA, in line with a previous study which suggested patients with more extensive length of SIAAD had greater false lumen (7).

The optimal treatment of SIAAD is currently inconclusive. In patients receiving conservative treatment, no death within 30-day occurred, similar to previous reports of 1% (13). All-cause mortality was 12.5% at long-term follow-up, and 37.5% patients needed aortic-related intervention, all of them were slightly higher than previous report (13, 14). Of the 23 patients who received surgical treatment, the 30-day all-cause mortality was 0%, consistent with previous reports of 0%–3% (13, 14).In addition, all-cause mortality at follow-up was 8.7%, and the incidence of aortic intervention was 4.3%, in line with previous reports of 5–11.4% and 6%–9.1%, respectively (13, 14, 17). A network meta-analysis found that conservative treatment was superior to open surgery and EVAR in terms of early mortality and late mortality (18). Su Sheng et al. reported that the adverse false lumen progression was higher for conservative treatment. Our study found that the all-cause mortality rate of conservative treatment was slightly but not significantly higher than that of surgical treatment. Furthermore, the adverse false lumen progression was significantly higher for conservative treatment than surgical treatment. Moreover, Mozes et al. (19) reviewed 41 cases of SIAAD and found that aortic rupture occurred in 14% cases overall. In a study that included 79% patients with SIAAD, aortic rupture was found in 10% cases, and all-cause mortality and complication were higher in patients who received conservative treatment (2). This suggests that SIAAD receiving conservative therapy remains at a higher risk of adverse progression and aortic rupture. Endovascular repair may promote aortic remodeling, reduce the risk of false lumen progression and decrease aortic rupture.

There were several limitations in this study. First, our cohort was a single-center retrospective study, which might increase selection bias. Second, the small sample size of the subgroups might decrease the statistical confidence.

## Conclusion

The clinical features of SIAAD vary depending on the location of the primary entry tear and the number of dissected aortic zones. Although surgical treatment was not associated with higher survival rate of SIAAD, it associated with a lower incidence of false lumen progression and a higher rate of aortic remodeling.

## Data Availability

The raw data supporting the conclusions of this article will be made available by the authors, without undue reservation.
