# Supplementary material for: Clinical characteristics and therapeutic strategy for patients with spontaneous isolated abdominal aortic dissection
Source: Front Cardiovasc Med. 2023 Aug 25;10:1214377. doi: 10.3389/fcvm.2023.1214377 (PMC10485829; doi:10.3389/fcvm.2023.1214377)
Supplement: Supplementary file 1 [file Table1.docx]

| Supplementary Table S1. The description of SVS/STS’s aortic zone in SIAAD | |
| --- | --- |
| Zone | **Range** |
| Zone 5 | Starting in aorta below the aortic fissure, proximal to the celiac origin |
| Zone 6 | From celiac origin to the top of superior mesenteric artery |
| Zone 7 | Starts in superior mesenteric artery origin, ends proximal to the origin of the highest renal artery |
| Zone 8 | Between the bilateral renal artery |
| Zone 9 | From the origin of the lowest renal artery to aortic bifurcation |
| Zone 10 | Common iliac artery |
| Zone 11 | External iliac artery |

| **Supplementary Table S2. Characteristics and management of endoleaks** | | | | |
| --- | --- | --- | --- | --- |
| **Case** | **Coexisting AAA** | **Type of endoleaks** | **Intraoperative management** | **Time to disappearance** |
| Case 1 | Y | Ia | balloon angioplasty | 3 days |
| Case 2 | Y | Ia | no treatment | 5 days |
| Case 3 | N | Ia | balloon angioplasty | 1 year |
| Case 4 | Y | Ib | Iliac extension stent+ balloon angioplasty | 3 days |
| Case 5 | N | Ib | no treatment | 1 month |
| Case 6 | N | II | no treatment | 1 year^#^ |
| Case 7 | Y | III | no treatment | 2 years |
| AAA, abdominal aortic aneurysm  ^#^ Ligation of the left internal iliac artery ligation was performed at 1 year to treat the endoleak. | | | | |
